# Chromosome comparison of 17 species / sub-species of African Goliathini (Coleoptera, Scarabaeidae, Cetoniinae)

**DOI:** 10.3897/CompCytogen.v10i2.8003

**Published:** 2016-06-03

**Authors:** Anne-Marie Dutrillaux, Bernard Dutrillaux

**Affiliations:** 1 UMR 7205 MNHN CNRS UMPC EPHE, Institut de Systématique, Evolution, Biodiversité. Muséum National d’histoire Naturelle, Sorbonne Universités, 57, rue Cuvier, CP39, 75005 Paris, France

**Keywords:** Cetoniinae, Goliathini, Coleoptera, chromosome banding, comparison

## Abstract

The mitotic karyotypes of 17 species of African Goliathini (Cetoniinae) are described using various chromosome banding techniques. All but one are composed of 20 chromosomes, mostly metacentric, forming a karyotype assumed to be close to that of the Polyphaga ancestor. The most derived karyotypes are those of *Goliathus goliatus* Drury, 1770, with eight pairs of acrocentrics and *Chlorocana africana* Drury, 1773, with only14 chromosomes. In species of the genera *Cyprolais* Burmeister, 1842, *Megalorhina* Westwood, 1847, *Stephanocrates* Kolbe, 1894 and *Stephanorrhina* Burmeister, 1842, large additions of variable heterochromatin are observed on both some particular autosomes and the X chromosome. Species of the genera *Eudicella* White, 1839 and *Dicronorrhina* Burmeister, 1842 share the same sub-metacentric X. Although each species possesses its own karyotype, it remains impossible to propose robust phylogenetic relationships on the basis of chromosome data only.

## Introduction

Cetoniinae, a large sub-family of Scarabaeidae (Coleoptera), is composed of about 3200 species grouped into ten tribes. Goliathini is one of the large tribes of this sub-family, with about 410 identified species, almost exclusively distributed in Asia and Africa. Data on their chromosome constitution are very scarce, with only three Asian species studied, *Rhomborrhina unicolor* Motschulsky, 1861 and *Rhomborrhina polita* Waterhouse, 1873 (Yadav et al. 1979), and *Jumnos ruckeri* Saunders, 1839 (Macaisne et al. 2006). Chromosome data are not much richer for the whole Cetoniinae sub-family, with only 28 species studied (Smith and Virkki 1978, Yadav et al. 1979, Macaisne et al. 2006, Dutrillaux et al. 2008). We also reported the chromosome formulae and NOR (Nucleolus Organizer Region) location of 14 additional species of Cetoniinae and described the karyotype of *Goliathus goliatus* Drury, 1770 (Dutrillaux and Dutrillaux 2012). All but four species had a 20,XY mitotic karyotype formula, and a Xyp meiotic sex chromosome formula in the males. In the literature, when chromosome morphology is provided, it appears that almost all autosomes are meta- or sub-metacentric, the X chromosome is generally acrocentric, with variable amounts of heterochromatin on its short arm and the Y is punctiform. These characteristics are shared with most Scarabaeoidea species studied (Yadav et al. 1979, Wilson and Angus 2004, 2005, Dutrillaux et al. 2011), which suggests that their karyotypes have not undergone drastic changes during evolution.

At first glance, this apparent karyotype homogeneity might indicate that chromosome rearrangements rarely occurred during the multiple speciation events having originated more than 30,000 Scarabaeoidea and 3200 Cetoniinae species. However, cautious comparisons, after chromosome banding and NOR localisation, revealed small differences in morphology, indicating that rearrangements, principally intra-chromosomal changes, have occurred (Wilson and Angus 2004, 2005, Dutrillaux et al. 2007, 2008). They are just difficult to detect because chromosome sizes are generally gradually decreasing and morphologies not so different, except when acrocentrics are formed. In addition, heterochromatin amounts may vary, independently of euchromatin rearrangements, which may cause variations of chromosome size and morphology not related to structural rearrangements *sensu stricto* and lead to misinterpretations.

Here, we report mitotic and meiotic chromosome data of 17 species of African species belonging to Goliathini. Their chromosomes are compared with the use of various staining techniques. Each species possesses its own karyotype. Most inter-specific differences seem to be the consequence of inversions and heterochromatin variations. With the exception of two species, *Goliathus goliatus* and *Chlorocana africana* Drury, 1773, all species conserved a karyotype composed of 20 chromosomes, principally sub-metacentric, thus not deeply different from that of many other Scarabaeidae.

## Material and methods

All but one species studied here were obtained by breeding developed by amateur entomologists from whom we obtained larvae. We pursued the breeding until imagine stage using oak leaf-mould. Diagnoses were performed according to Sagai and Nakai (1998). Young male imagines were anaesthetized with ethyl acetate and dissected for extracting their gonads. Testes were dropped and dilacerated in O.88 M KCl where they remained for 15 min (metaphase stages studies) or 7h (pachytene stage analysis). The cell in suspension were transferred into either O.55 M KCl or diluted fetal calf serum (1/3 serum: 2/3 distilled water) for 15 min., fixed and spread as described by Dutrillaux et al. (2010). Giemsa staining and C-banding were successfully applied in all species and silver (NOR) staining in meiotic cells of all but two species. For some species, we used light Giemsa (LG = low Giemsa stain concentration and short staining time) to improve chromatid differentiation and obtain a kind of G-banding on pro-metaphases from spermatogonia or gonocytes. We also occasionally used cells from the mid gut, according to Angus (1982). Only the karyotypes of *Dicronorrhina derbyana oberthuri* Deyrolle, 1876 and Goliathus goliatus have been described before, but we reported formulae and NOR localization for all these species (Dutrillaux and Dutrillaux 2012). We classified their chromosomes by decreasing size, as usual, but making abstraction of heterochromatin, present in large and variable amounts in some species, and followed ISCN (1985) chromosome nomenclature.

## Results and discussion

### Brief description of the male karyotypes

**Figures 1–6.**
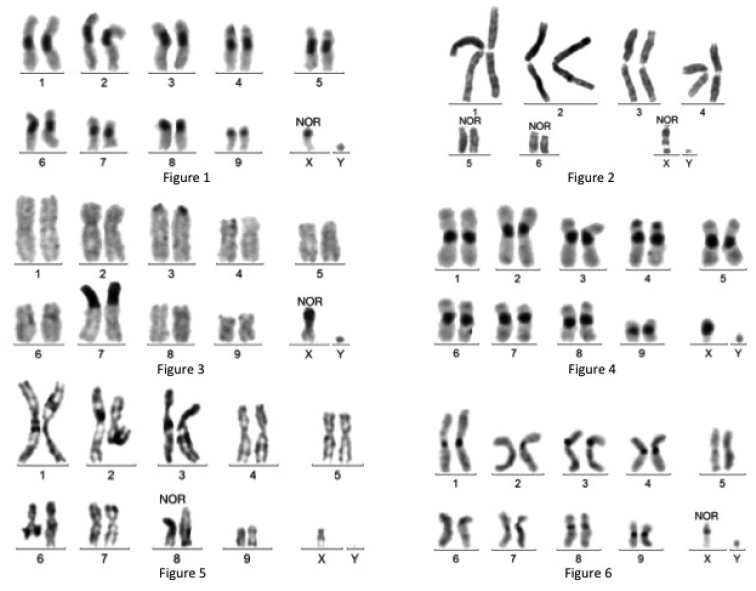
**1** C-banded karyotype of *Amaurodes passerini* **2** Giemsa stained karyotype of *Chlorocana africana* **3** C-banded karyotype of *Cyprolais hornimani*, with large heterochromatic fragments on chromosomes 7 and X **4** C-banded karyotype of *Dicronorrhina derbyana derbyana* **5** Giemsa stained karyotype of *Dicronorrhina micans* **6** C-banded karyotype of *Eudicella aethiopica*.

***Amaurodes passerini*** Westwood, 1844 (Fig. 1): mitotic formula: 20,XY; meioformula: 9+Xyp. - Chromosome morphology: pair no 1 metacentric; pairs no 2–7 sub-metacentric; pairs no 8 and 9 and X acrocentric; Y punctiform.

- C-banding: fairly large juxta-centromeric C-band on pairs 1-8, smaller on pair 9 and X, absent on the Y. Presence of a faint C-band at the telomeric region of the chromosome 4p arm (4pter).

- After LG staining, a banding differentiates all chromosome pairs (Fig. 18).

- Silver staining: at pachynema of meiotic prophase, nucleoli are always next to the sex bivalent; at metaphase I, strong staining of the space between the X and Y. NOR location: Xp (p=short arm, according to ISCN, 1985)).

***Chlorocala africana*** Drury, 1773 (Fig. 2): mitotic formula: 14,XY; meioformula: 6+Xyp.

- Chromosome morphology: pairs N°1-4 metacentric; pairs N° 5 and 6 acrocentric; X acrocentric with two frequent gaps and Y punctiform.

- C-banding: quite discrete at all centromeric regions, and also at intercalary regions of chromosomes 1–4.

- Silver staining: present at mitotic metaphase on short arms of acrocentrics (N° 5, 6 and X). At pachynema, the sex bivalent is intensely stained, as well as the centromeric regions of bivalents 5 and 6. Nucleoli are associated with the sex bivalent and bivalent 6 short arm. At metaphase I, there is an intense staining of the space between the X and Y. NOR location: Xp, 5p, 6p.

***Cyprolais hornimani*** Bates, 1877 (Fig. 3): mitotic formula: 20,XY; meioformula: 9+Xyp.

- Chromosome morphology: all the autosomes but N° 3 appear to be meta- or sub-metacentric after Giemsa staining. The X is sub-metacentric and the Y punctiform.

- C-banding: very faint or absent on most autosomes. Only the acrocentric N° 3 is clearly C-banded at centromeric region. Large additional heterochromatic segments are present at 7p terminal region and on Xp.

- Silver staining: intense on the Xp arm and on the Y at mitotic metaphase. At pachynema, nucleoli are alongside the sex bivalent. The heterochromatic region of bivalent 7 is frequently close or at contact with the sex bivalent. At metaphase I, intense staining of the space between the X and Y. NOR location: Xp, Y?

***Dicronorrhina derbyana derbyana*** Westwood, 1843 (Fig. 4) and ***Dicronorrhina derbyana oberthuri*** Deyrolle, 1876: mitotic karyotype formula 20,XY; meioformula : 9+Xyp. One male *Dicronorrhina derbyana oberthuri* with a disomy Y was reported (Dutrillaux and Dutrillaux 2011b). Besides this particularity, no difference was noticed between the two subspecies.

- Chromosome morphology: all the autosomes are meta- or sub-metacentric; the X is metacentric and the Y punctiform.

- C-banding: large juxta-centromeric C-bands on centromeric regions of all chromosomes, including the X and Y, with only slight variations. Discreet C-bands are present at terminal or sub-terminal regions of the 4p arm, and occasionally other chromosome arms.

- LG staining: a discreet banding differentiates all chromosome pairs.

- NOR staining: at pachynema, nucleoli are located alongside the short arm of a small bivalent, N° 7 or 8.

***Dicronorrhina micans*** Drury, 1773 (Fig. 5): mitotic formula: 20,XY; meioformula: 9+Xyp.

- Chromosome morphology: all autosomes but pair N° 8 are meta- or sub-metacentric. Pair N° 8 is acrocentric, the X is sub-metacentric and the Y punctiform.

- C-banding: large juxta-centromeric bands in pairs N° 1-7, smaller in pairs N° 8 and 9 and sex chromosomes.

- Silver staining: at pachynema, nucleoli are located on the short arm of bivalent N° 8. NOR location: 8p arm

***Eudicella aethiopica*** Müller, 1941 (fig. 6): mitotic formula: 20,XY; meioformula: 9+Xyp.

- Chromosome morphology: all autosomes are meta- or sub-metacentric. The X is sub-metacentric and the Y punctiform.

- C-banding: fairly large juxta-centromeric C-bands on pairs N° 1, 3, 4 and 9, small on pairs 2, 7, 8 and X, and very small on pairs N° 5 and 6 and Y.

- Silver staining: intense on the Xp arm and the Y at mitotic metaphase; presence of nucleoli in association with the intensely stained X component of the sex bivalent at pachynema; and intense at the X and Y junction of the Xyp bivalent at metaphase I. NOR location: Xp arm.

**Figures 7–12.**
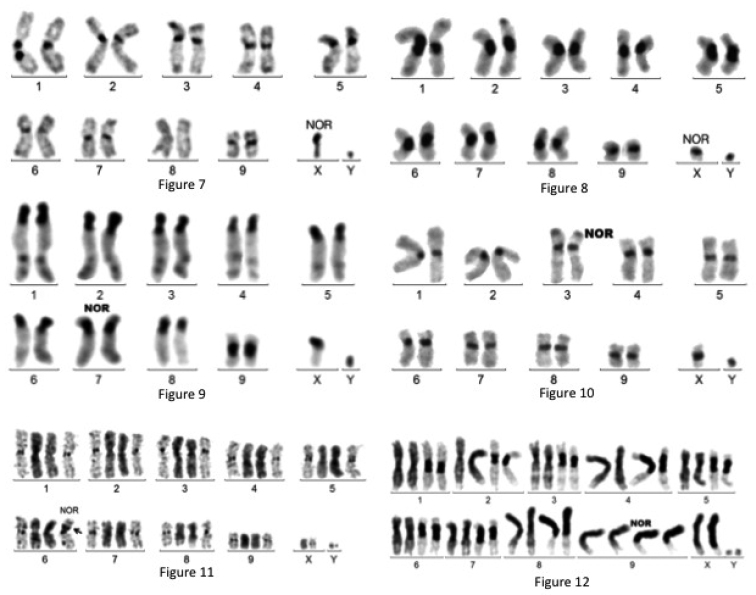
**7** C-banded karyotype of *Eudicella gralli* **8** C-banded karyotype of *Eudicella smithi*. NOR on Xp arm **9** C-banded karyotype of *Goliathus goliathus*. NOR on 7 p arm **10** C-banded karyotype of *Mecynorrhina polyphemus confluens*. NOR on 3p arm **11** Giemsa stained (center) and C-banded karyotype of *Mecynorrhina torquata*. NOR on 6p arm **12** Giemsa stained (left) and C-banded (right) karyotype of *Megalorrhina harrisi*. The short arms of chromosomes 8, 9 and X are entirely heterochromatic. NOR on 9p arm.

***Eudicella gralli*** Buquet, 1836 (Fig. 7): mitotic formula: 20,XY; meioformula 9+Xyp.

- Chromosome morphology: all autosomes meta- or sub-metacentric; X acrocentric or sub-metacentric (inter-individual variation?), Y almost punctiform.

- C-banding: large C-bands at all juxta-centromeric regions, except for pair N° 8 and chromosome Y. Frequent small C-band on chromosome 3pter. The Xp arm may be either C-banded (acrocentric form) or not (sub-metacentric form).

- Silver staining: as for *Eudicella aethiopica*: Xp arm.

***Eudicella smithi*** MacLeay, 1838 (Fig. 8): mitotic formula: 20,XY; meioformula: 9+Xyp.

- Chromosome morphology: all autosomes and the X meta- or sub-metacentric and the Y punctiform.

- C-banding: large and variable juxta-centromeric C-bands on pairs N° 1–8, smaller on pair N° 9 and sex chromosomes. A dispensable C- band exists on chromosome 2pter.

- NOR staining: as for *Eudicella aethiopica*: Xp arm.

***Goliathus goliatus*** Drury, 1770 (Fig. 9): mitotic formula: 20,XY; meioformula 9+Xyp. The C-banded karyotype was reported in Dutrillaux and Dutrillaux (2012).

- Chromosome morphology: the X chromosome and all autosomes but pair no 9 are acrocentric. The Y is punctiform.

- C-banding: intense C-bands are present at the centromere regions of most chromosomes. Faint C-bands are also distally located on the long arms of chromosomes1 to 5.

- LG staining: all chromosome pairs could be identified.

- NOR staining: At pachynema, the sex bivalent is intensely stained, and nucleoli are associated with the short arm of an acrocentric, presumably bivalent 7. At metaphase I, the space between the X and Y is intensely stained. NOR location: 7p arm.

***Mecynorrhina polyphemus confluens*** Fabricius, 1781 (Fig. 10): mitotic formula: 20,XY; meioformula: 9+Xyp.

- Chromosome morphology: all autosomes meta- or sub-metacentric; X metacentric, Y punctiform.

- C-banding: fairly intense at all juxta-centromeric regions of all autosomes and sex chromosomes; presence of a C-band on 3pter.

- LG staining: all chromosome pairs could be identified (Fig. 18).

- Silver staining: at pachynema, nucleolus are alongside the terminal region of bivalent 3; intense staining of the sex bivalent in the space between X and Y at metaphase I. NOR location: 3p arm.

***Mecynorrhina torquata*** Drury, 1782 (Fig. 11): mitotic formula: 20,XY; meioformula: 9+Xyp.

- Chromosome morphology: all autosomes meta- or sub-metacentric; X metacentric, Y small.

- C-banding: moderately large C-bands at juxta-centromeric regions of pairs N° 1–4 and 6–8, small on pairs 5 and 9, and almost inexistent on sex chromosomes; presence of a small C-band on 3pter.

- LG staining: identification of all chromosome pairs.

- Silver staining: intense at the proximal region of chromosome 6p arm at mitotic metaphase and on the Xyp bivalent at metaphase I. NOR location: 6p arm.

***Megalorrhina harrisi*** Westwood, 1847 (Fig. 12): mitotic formula: 20,XY; meioformula 9+Xyp. The parachute like sex bivalent is unusually large, with the presence of heterochromatin, opposite to the Y, which is associated with the euchromatic part of the X.

- Chromosome morphology: all the autosomes and the X appear to be meta- or sub-metacentric after Giemsa staining, and the Y is quite small.

- C-banding: large or very large juxta-centromeric C-bands on all chromosomes but the Y. On pairs N° 8 and 9 and the X, one arm is entirely heterochromatic. Thus, these chromosomes must be regarded as acrocentric, although they look metacentric. Large variations of heterochromatin lead to a marked polymorphism. For instance, the size of the X may vary by twofold among individuals.

- LG staining: identification of all chromosome pairs (Fig. 18)

- Silver staining: at pachynema, the sex bivalent is intensely stained, and nucleoli are at contact with the short arm of bivalent 9. At metaphase I, the space between X and Y is stained, as usual in Xyp bivalents. NOR location: 9 p arm

**Figures 13–17.**
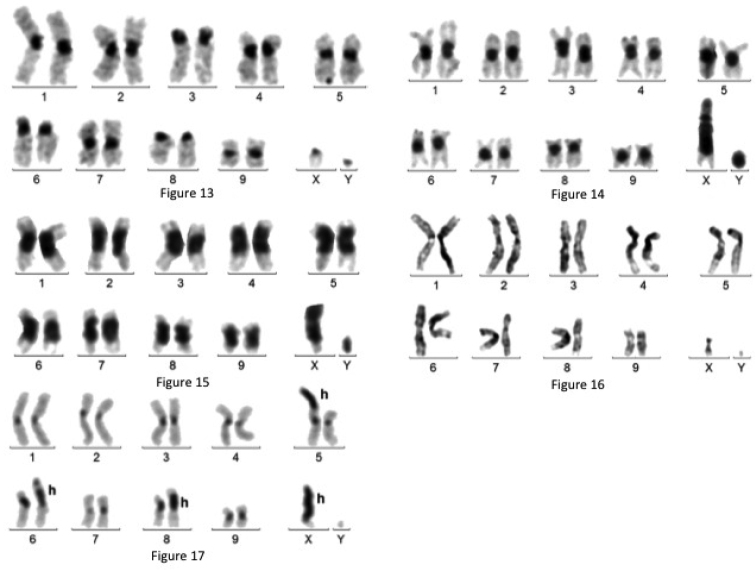
**13** C-banded karyotype of *Plaesiorrhinella watkinsiana* **14** C-banded karyotype of *Rhamphorrhina bertoloni*. Heterochromatic Xp arm **15** C-banded karyotype of *Stephanocrates preussi*. Heterochromatic Xp arm **16** Giemsa stained karoytype of *Stephanorrhina guttata* **17** C-banded karyotype of *Stephanorrhina princeps*. H : heterochromatin.

***Plaesiorrhinella watkinsiana*** Lewis, 1879 (Fig. 13): mitotic formula: 20,XY; meioformula: 9+Xyp.

**Figure 18.**
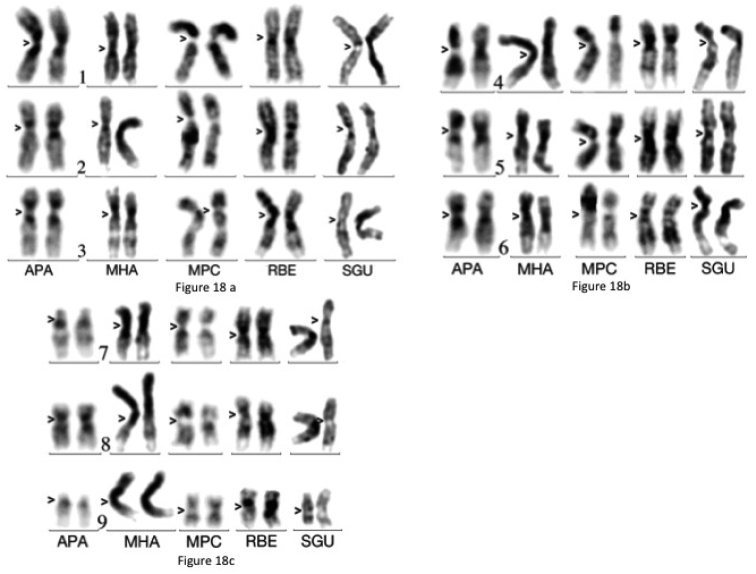
Comparison of autosomes from gonocytes of 5 species after Giemsa light staining: *Amaurodes passerini* (APA), *Megalorrhina harrisi* (MHA), *Mecynorrhina polyphemus confluens* (MPC), *Rhamphorrhina bertoloni* (RBE) and *Stephanorrhina guttata* (SGU). **a** Chromosomes 1–3 **b** chromosomes 4–6 **c** chromosomes 7–9. Centromeres are indicated by arrow heads.

- Chromosome morphology: pairs N° 1, 2, 4, 5, 7 and 9 meta- or sub-metacentric, pairs N° 3, 6 and 8 acrocentric. The X is acrocentric and the Y punctiform.

- C-banding: fairly intense at all juxta-centromeric regions of all autosomes and faint on sex chromosomes.

- LG staining: identification of all chromosome pairs.

- Silver staining: at pachynema, nucleoli are associated with the centromeric region of the acrocentric bivalent 6. NOR location: 6p arm.

***Rhamphorrhina bertolonii*** Lucas, 1879 (Fig. 14): mitotic formula: 20,XY; meioformula: 9+Xyp.

- Chromosome morphology: all autosomes are metacentric or sub-metacentric, the X is a large sub-metacentric, and the Y is a small metacentric.

- C-banding: limited to centromeric regions on autosomes, it stains most of the X and Y.

- LG staining: identification of all chromosome pairs (Fig. 18).

- NOR staining: at pachynema, nucleoli are located alongside the sex bivalent, which is unusually large, due to the presence of a large heterochromatic fragment on the X chromosome. This heterochromatin prevents to accurately locate both centromere and NOR on this chromosome.

***Stephanocrates preussi*** Kolbe, 1892 (Fig. 15): mitotic formula: 20,XY; meioformula: 9+Xyp.

- Chromosome morphology: all the autosomes are meta- or sub-metacentric. The X is unusually large and sub-metacentric; the Y is acrocentric.

- C-banding: very large C-bands at all juxta-centromeric regions of all chromosomes, representing 30-40% of their whole length. The large size of the X is principally related to the presence of heterochromatin. X and Y form a large parachute bivalent at metaphase I.

- Silver staining: at pachynema, nucleoli are recurrently located near the centromere region of a large metacentric, which could not be identified. At metaphase I, the large parachute sex bivalent is deeply stained between the X and the Y. NOR location: autosomal.

***Stephanorrhina guttata*** Olivier, 1789 (Fig. 16): mitotic formula: 20,XY; meioformula: 9+Xyp

- Chromosome morphology: all the autosomes and the X appear to be metacentric or sub-metacentric (chromosomes 1, 3, 6 and 8) or sub-metacentric after Giemsa staining, and the Y is quite small.

- C-banding: in addition to non-remarkable juxta-centromeric C-bands, presence of a small C-band on the terminal region of the 5p arm. The small Xp arm is heterochromatic and the Y remains unstained.

- LG staining: identification of all chromosome pairs (Fig. 18).

- Silver staining: nucleoli remain alongside the sex bivalent at pachynema and the space between X and Y is intensely stained at metaphase I. NOR location: probably on the heterochromatic short arm of the X.

***Stephanorrhina princeps*** Oberthür, 1880 (Fig. 17): mitotic formula: 20,XY; meioformula: 9+Xyp.

- Chromosome morphology: all the autosomes and the X appear to be metacentric or sub-metacentric after Giemsa staining, and the Y is quite small.

- C-banding: in addition to juxta-centromeric heterochromatin on all chromosomes, large additional and polymorphic heterochromatic segments occur on chromosomes 5,6, 8 and the X.

- LG staining: identification of all chromosome pairs.

## Chromosome comparison and evolution

The karyotypes of all species except *Chlorocana africana* are composed of 20 chromosomes, a number observed in most Cetoniinae, Dynastinae and Melolonthinae (Smith and Virkki 1978, Dutrillaux et al. 2007, 2008, 2011, Gianoulis et al. 2011) and other Scarabaeidae species studied so far (Angus et al. 2007, Bione et al. 2005, Dutrillaux and Dutrillaux 2013, Silva et al. 2009, Moura et al. 2003, Smith and Virkki 1978, Wilson and Angus 2004, 2005, Yadav et al. 1979). As proposed by several of these authors, it is very likely that this number is that of their common ancestral karyotype. Thus, the karyotype of *Chlorocana africana*, with 14 chromosomes is highly derived. It possesses three pairs of very large chromosomes, which probably originated by translocation (fusion) of ancestral chromosomes.

The 16 other karyotypes comprise nine pairs of autosomes of gradually decreasing size. Their similar sizes among the different karyotypes may have the following interpretations:

– neither translocations nor other inter-chromosomal exchanges occurred during evolution/speciation processes;

– exchanges occurred, but involved very small fragments, hard to detect;

– exchanges involved large fragments of similar sizes, preserving chromosome size.

Exchanges of very small fragments are unlikely. They are harmful because they lead to deleterious, but viable chromosomal imbalances, in progeny of heterozygote translocation carriers, as shown in human pathology. Thus, they should have been strongly counter-selected during evolution. Exchanges of large fragments may exist, but it would be very unlikely that they systematically involved fragments of similar size. Thus, the more likely interpretation is that speciation and evolution processes have occurred with few or without inter-chromosomal rearrangements in this tribe of beetles. Then, either chromosome rearrangements rarely occurred, or they were mostly of the intra-chromosomal type such as inversions. This last interpretation is, by far, the most likely (Dutrillaux and Dutrillaux 2009). Unfortunately, the occurrence of inversions is not easy to detect in poorly banded chromosomes, as those of beetles, except when they drastically modify their morphology, as changing a meta-/sub-metacentric into an acrocentric, or *vice versa*. In beetles, most autosomes are metacentric or sub-metacentric. Unless there is a strong selective constraint against acrocentrics, it is likely that ancestral karyotypes were already composed of meta- and sub-metacentrics. Starting from such a karyotype, the presence of acrocentrics would sign the occurrence of inversions. Acrocentrics are observed in six species. As regards their numbers, the karyotype of *Goliathus goliatus*, with eight acrocentric pairs, is by far the most derived. The five other karyotypes exhibit one to three acrocentric pairs. Most chromosomes involved are small. Although some recurrences exist, principally for chromosome 8, which is acrocentric in *Amaurodes passerini*, *Dicronorrhina micans*, *Goliathus goliathus*, *Megalorrhina harrisi* and *Plaesiorrhinella watkinsiana*, this does not allow us to propose a phylogenetic scheme based on the sequence of autosome inversions.

The X chromosome exhibits four different morphologies: acrocentric, sub-metacentric, metacentric and more or less sub-metacentric, with one euchromatic and another heterochromatic arm (C-banded). The two former morphologies were observed in species of Cetoniini, Dynastinae and Melolonthinae (Dutrillaux et al. 2007, 2008, Gianoulis et al. 2011) but it was proposed that the acrocentric was likely to be the ancestral form. Thus, the five species with a sub-metacentric X (two *Dicronorrhina* and three *Eudicella* species) might have a same derivative X, which may constitute an argument to put together the two genera. The two species with a metacentric X belong to the same genus *Mecynorrhina*.

Large amounts of heterochromatin are present in the X chromosome of six species belonging to five genera (*Cyprolais*, *Rhamphorrina*, *Megalorrhina*, *Stephanocrates* and *Stephanorrhina*). This is also an argument to put together these five genera. Finally, the presumably ancestral acrocentric X is conserved in 4 species: *Amaurodes passerini* and *Plaesiorrhinella watkinsiana* and 2 with highly rearranged autosomes, *Goliathus goliatus* and *Chlorocana africana*.

As in many other Scarabaeidae (Dutrillaux and Dutrillaux 2012), the NOR is frequently located on the X. In species with autosomal NORs, the NOR is located on different autosomes, which suggests that different events displaced it from the ancestral position on the X chromosome.

On the whole, it remains very difficult to propose undisputable phylogenetic relationships by using these classical cytogenetic data. The detected rearrangements are too few, and the species studied probably represent a too small and heterogenous sample of the sub-family. If inversions are in cause, it is not certain that molecular cytogenetics (FISH) would significantly improve the results, at least by the use of chromosome painting.

Finally, an improvement could come from chromosome banding, but a major problem remains: the difficulty for inducing a consistent banding of euchromatin. Beetle chromosomes are apparently not composed of large heterogeneous DNA fragments, as ALU and LINE sequences, associated to R- and G-banding in mammalian chromosomes (Bickmore and Craig 1997). We tried to use various techniques of DNA denaturation (Dutrillaux and Lejeune 1971), without success. We also tried enzymatic digestions, which inconsistently gave some results (Dutrillaux et al. 1971, Seabright 1971). To our knowledge, euchromatin banding of mitotic chromosomes was rarely induced in beetles (Angus 1982), which illustrates a technical difficulty. In this study, we tried to take advantage of the variations of chromosome compaction from cell type to cell type, and in a given cell type, with cell differentiation level. Drastic changes of chromatid compaction occur during early gametogenesis, in relation with variations of DNA methylation, around the birth period of the mouse (Coffigny et al. 1999, Bernardino-Sgherri et al. 2002). The particular aspects of chromosomes, described in mouse gonocytes, are occasionally observed in immature gonads of beetles. In particular, some germ cells, probably gonocytes, exhibit a poor banding that appropriate Giemsa staining and computer driven contrast adjustments can improve. This allowed us to perform more accurate comparisons for five species (Fig. 18), and show the occurrence of inversions among sub-metacentric chromosomes. These inversions group *Amaurodes passerini* and *Megalorrhina harrisi* on the one hand and *Rhamphorrhina bertoloni* and *Stephanorrhina guttata* on the other hand. *Megalorrhina polyphemus* appears to have an intermediary position. This confirms that apparently similar karyotypes can differ by cryptic chromosome rearrangements, as shown in many mammalian species, but not in beetles.

## Conclusion

All the karyotypes of the 17 studied species differ from each other in some respects by inversions, heterochromatin variations and translocations. As expected, congeneric species possess more similar karyotypes than species from different genera, but it remains impossible to propose a phylogeny based on chromosome changes. It is noteworthy that *Goliathus goliathus*, which has some remarkable phenotypic characters, such as large size, hairy thorax, cephalic horns in the male, has a most derived karyotype, with 8/9 inverted autosomes. It would be tempting to consider that a relationship, even indirect, exists between the accumulation of chromosome rearrangements and that of gene mutations determining phenotype changes. However, *Chlorocana africana*, which has a non-remarkable morphology among Cetoniinae, but a highly rearranged karyotype, confirms that it would be hazardous to propose such correlation.
